# Supplementary material for: Expanded Newborn Screening for Inborn Errors of Metabolism at a Single Center in Louisiana (2005–2024): Outcomes
Source: Int J Neonatal Screen. 2025 Dec 9;11(4):112. doi: 10.3390/ijns11040112 (PMC12734074; doi:10.3390/ijns11040112)
Supplement: Supplementary file 1 [file IJNS-11-00112-s001.zip › Supplemental Table S1.pdf]

Supplemental Table S1. Clinical outcome of metabolic diseases

Amino acidopathy (14)	
HT1 (5)	Five patients (one male, four females) from four families were diagnosed with HT1, with ages ranging from 3y11m to 14y11m. The average time to NBS result was 8.8 days. Four patients started NTBC and dietary protein restriction between days 18–20 of life. One patient, identified early due to a positive family history, began treatment on day 5, following early biochemical testing. All patients demonstrated normal growth and development, with no signs of liver disease or Fanconi syndrome. One patient had bilateral nephrolithiasis.
MSUD (4)	Four patients (three females, one male) were diagnosed with classic MSUD, with ages ranging from 6y6m to 12y11m: Patient 1 (12y11m), Patient 2 (12y9m), Patient 3 (7y2m), and Patient 4 (6y6m).<br>Patients 1 and 4 were symptomatic at diagnosis, with elevated branched-chain amino acids (BCAAs) on plasma analysis, requiring critical care hospitalization with continuous renal replacement therapy (CRRT), IV dextrose, and dietary management.<br>Patient 2 showed elevated leucine at the time of diagnosis but remained stable with outpatient dietary management.<br>Patient 3 was asymptomatic at diagnosis and did not require intensive care.<br>Patient 1 had maintained good metabolic control through dietary management, with normal development and strong academic performance. Patient 2 has remained stable without metabolic decompensation since age 6, with normal growth and development. Patients 3 and 4 underwent liver transplantation around age 5 due to recurrent metabolic crises; both have exhibited developmental delays.
HCU (3)	Three patients (one female, two males) were diagnosed with HCU: Patient 1 (14y), Patient 2 (6y9m), and Patient 3 (6y). All received early treatment with dietary protein restriction, metabolic formula, betaine, and pyridoxine. Patient 2 responded well to pyridoxine, while Patient 3 did not tolerate it and Patient 1 didn't respond.<br>Patient 1 had an absence seizure at age 7, resolved by age 8. Genetic testing showed compound heterozygous pathogenic *CBS* variants: c.362G>A (p.Arg121His*)* and c.667-(14_7)del18. Despite poor metabolic control (5-year mean total homocysteine: 204 ± 30.5 µmol/L), she has normal intelligence and performs well academically.<br>Patient 2, with cerebral palsy and global developmental delay of unknown cause, had *CBS* variants c.1330G>A (p.Asp444Asn*)* and c.738delG (p.Lys247Serfs22)*. Exome sequencing revealed no other diagnoses. He achieved good metabolic control with pyridoxine (mean homocysteine: 36.4 ± 24 µmol/L).

	Patient 3, with normal growth and development, has CBS homozygous pathogenic variant c.572C>T (p.Thr191Met). He showed good response to treatment (mean homocysteine: 69 ± 43 µmol/L).<br>None of the patients exhibited ocular, vascular, or skeletal complications.
MAT1 deficiency (1)	A 6y11m old male was diagnosed with MAT I deficiency. His mean ± SD plasma methionine level was 495 ± 314 µmol/L. Despite being on a normal diet, levels never exceeded 800 µmol/L. Growth and development have been normal.
PTPS deficiency (1)	A 3y9m old female was diagnosed with PTPS deficiency after elevated phenylalanine was detected on NBS. She was started on tetrahydrobiopterin (BH_4) at diagnosis and has been followed by Neurology for treatment with L-Dopa/carbidopa and 5-hydroxytryptophan (5-HTP). Her growth and development have been normal.
Urea cycle defects (7)	
Citrullinemia type 1 (5)	Five patients (3 females, 2 males) were diagnosed with Citrullinemia Type I, with ages ranging from 3 to 19y5m. Four (Patients 1, 2, 3, and 5) were asymptomatic when NBS results became available (days 5–8 of life). One (Patient 4) presented with hyperammonemia on day 6, coinciding with the NBS result. All patients began dietary protein restriction, ammonia scavengers, and arginine supplementation at diagnosis.<br>Patient 1 (19y5m): Mean citrulline 1408 ± 329 µmol/L. No hyperammonemia since age 8. Normal growth and development.<br>Patient 2 (14y3m): Mean citrulline 253 ± 45 µmol/L. Lost to follow-up since age 8. No hyperammonemia. Normal development at most recent visit.<br>Patient 3 (9y1m): Mean citrulline 604 ± 80 µmol/L. No hyperammonemia. Normal growth and development.<br>Patient 4 (4y6m): Mean citrulline 3236 ± 665 µmol/L. Two hyperammonemic episodes (newborn period and age 3). Developmental delay and behavioral concerns noted.<br>Patient 5 (3y): Mean citrulline 93 ± 26 µmol/L. Asymptomatic, no hyperammonemia. Normal growth and development.
ASA (2)	Two patients (one female, one male) were diagnosed with ASA (8y7m) and 2 (12m). Patients 1 and 2 developed symptomatic hyperammonemia (293 µg/dL and 601 µg/dL on days 7 and 5, respectively) before NBS results were available (days 7 and 13). Both began ammonia scavengers, arginine, and dietary management at diagnosis and have had no further hyperammonemic episodes.<br>Developmentally, Patient 1 has mild delays and learning difficulties; Patient 2 has global developmental delays.
Organic acidemia (43)	
GA1 (18)	Eighteen patients (11 females, 7 males) from four families, aged 19m to 17y5m, were diagnosed with glutaric aciduria type I (GA1). All were high excretors and received prompt

	treatment, including dietary management, metabolic formula, and carnitine supplementation.<br>Two patients experienced neurological injury following encephalopathic crises at 7m and 1y of age (now aged 7y5m and 8y9m). The first, born prematurely at 33 weeks with cleft palate, dysmorphic features, atrial septal defect, and feeding difficulties requiring G-tube, had global developmental delays and failure to thrive. Exome sequencing did not reveal additional genetic diagnosis. The second had normal development until an unmonitored acute illness led to a crisis. Both now have severe motor dysfunction.<br>Eight patients have normal development without additional medical concerns. Of the remaining eight: one has learning disability, language and fine motor delays; one has a learning disability; and three have ADHD. Clinical status is unknown for three patients.
IVA (9)	Nine patients (four females, five males), aged 16m to 15y11m, were diagnosed with IVA. Eight were asymptomatic at the time of NBS result availability, with a mean ± SD turnaround time of 16.7 ± 13 days (data unavailable for one patient). All eight had mild IVA, with a mean ± SD C5 level of 2.75 ± 1.2 nmol/mL.<br>Initial management included metabolic formula, dietary protein restriction, and carnitine supplementation. None experienced metabolic decompensation, and dietary restrictions were later liberalized. All showed normal growth and development, except for one patient with language delay and autistic features, despite stable metabolic status.
MMA (7)	Total of seven patients were diagnosed with MMA, including four males and three females. Their ages are as follows: Patient 1 (19y7m), Patient 2 (16y9m), Patient 3 (16y7m), Patient 4 (15y5m), Patient 5 (11y8m), Patient 6 (5y5m), and Patient 7 (2y4m). Six had MMA-mut; one (Patient 4) had MMA-CblB.<br>Patient 1 was diagnosed and treated shortly after birth due to a positive family history but still experienced neonatal metabolic decompensation. Patients 4 and 5 were asymptomatic when NBS results became available on days 5 and 6. Patients 3, 6, and 7 developed symptoms before NBS results, on days 6, 9, and 1, respectively. All patients received early treatment, including dietary protein restriction, metabolic formula, and carnitine supplementation. Despite this, all experienced multiple episodes of metabolic decompensation.<br>Patients 1–4 underwent combined liver and kidney transplantation at ages 13, 15, 15, and 14, respectively:<br>Patient 1: Global developmental delay, optic nerve atrophy, visual impairment, short stature, basal ganglia stroke at age 4 with spastic diplegia, ESRD pre-transplant.<br>Patient 2: Short stature, ESRD; no metabolic decompensation for over five years pre-transplant; cognitively normal.<br>Patient 3: Language delay, bilateral congenital cataracts, profound SNHL, short stature.

	Patient 4: Intellectual disability, progressive visual loss, short stature, recurrent pancreatitis, lactic acidosis, and CKD stage 3.<br>Patients 5 and 6 suffered basal ganglia strokes at ages 5 and 4, respectively. Patient 5 had normal development prior to the stroke; Patient 6 was diagnosed with language delay and autism before his stroke.<br>Patients 5, 6, and 7 experienced 6, 3, and 5 metabolic decompensations, respectively, within the past year.
Cobalamin C deficiency (4)	Four patients (two females, two males) were diagnosed with cobalamin C (cblC) deficiency: Patient 1 (11y), Patient 2 (6y7m), Patient 3 (4y7m), and Patient 4 (3y4m).<br>All patients were treated with hydroxocobalamin, betaine, and carnitine, and had normal height and head circumference. Patients 1 received care out-of-state.<br>Developmental delays were noted in all cases:<br>Patient 1 (mean±SD total Hct 28.6±2.5 µmol/L): Intellectual disability, autism, epilepsy, unilateral hearing loss, and cardiomyopathy.<br>Patient 2 (mean±SD total Hct 15.9±2.5 µmol/L): Multiple congenital anomalies of unknown etiology, including anotia, mixed hearing loss, retrognathia, bronchial anomaly, facial palsy, lagophthalmos, G-tube dependency, and severe language delay.<br>Patients 3 (mean±SD total Hct 44.7±22 µmol/L) and 4 (mean±SD total Hct 58.8±64 µmol/L): Language delay.
PA (4)	Four male patients from three families were diagnosed with PA, with ages ranging from 13y6m to 17y11m. All were asymptomatic at diagnosis and promptly started on dietary protein restriction, metabolic formula, and carnitine supplementation.<br>Patients 1 and 2 (siblings) have had a mild disease course with no episodes of metabolic decompensation and are performing well academically.<br>Patient 3 has developmental delay and autism but no history of metabolic crises.<br>Patient 4 has a history of epilepsy, ADHD, and a learning disability. He has experienced frequent hospitalizations for intravenous dextrose during episodes of illness, with approximately one episode of metabolic decompensation per year. Brain MRI findings have been normal.<br>All patients have normal growth, cardiac evaluations, and no signs of optic disc atrophy.
HLCSD (1)	One patient was diagnosed with HLCSD prior to the availability of NBS results, which were reported on day 5 of life. She was admitted to the NICU on day 1 for hypothermia, seizures, and worsening metabolic acidosis. High-dose biotin (30 mg/day) and carnitine supplementation were initiated upon diagnosis. The patient had global developmental delay and, at 21 months of age, died from intractable metabolic acidosis triggered by a gastrointestinal infection, despite intensive treatment including continuous renal replacement therapy (CRRT).

Fatty acid oxidation disorders (115)	
VLCADD (30)	Thirty patients (12 females, 18 males), age ranging from 9m to 19y6m, were diagnosed with VLCADD. The majority had a mild disease course. Twenty-nine patients have not experienced significant metabolic decompensation or cardiac/liver involvement. One patient developed transient muscle symptoms after strenuous exercise, with a peak CK of 1200 U/L that later normalized. Another patient died during an intercurrent illness, the established protocols for treatment were not followed during an intercurrent illness resulting in the child dying at 13 months old.
MCADD (74)	Seventy-four patients were diagnosed with MCADD. To the best of our knowledge, three patients with MCADD have died.<br>Case 1: The patient died on the first day of life due to fetal hydrops with severely hypoplastic lungs. Newborn screening results became available on day 4 of life.<br>Case 2: The patient, a sibling of Case 1, died on the third day of life of unknown cause. NBS results became available on day 5 of life.<br>Case 3: The patient died of unknown cause at an unspecified age. The diagnosis of MCADD was made via NBS, with results available on day 13 of life. The patient was lost to follow-up after 3 months of age.
MADD (5)	Five patients (one male, four females), ages ranging 3 m to 5y5m, were diagnosed with MADD.<br>Patient 1 died in the first week of life before NBS results were available. Although medical records are unavailable, urine organic acid analysis on day 2 strongly suggested MADD. No molecular testing was performed. This case was likely severe neonatal-onset MADD.<br>Patient 2 (5y5m) had abnormal NBS and urine organic acids consistent with MADD but was lost to follow-up before confirmatory molecular testing.<br>Patient 3 died at age 2 following recurrent metabolic decompensation, dilated cardiomyopathy, and congestive heart failure. She presented with recurrent episodes of hypoglycemia, elevated liver enzymes and CK, hypotonia, and global developmental delay. Treatment included protein restriction, high-carb diet, riboflavin, beta-hydroxybutyrate, and carnitine.<br>Patient 4 (2y6m) remains asymptomatic with normal labs and echocardiogram. He is not on treatment.<br>Patient 5 (3m) had elevated acylcarnitines on NBS, with plasma levels of C8, C10, and C12 above reference range. Urine organic acids and acylglycines were normal. Molecular testing revealed compound heterozygous VUS in *ETFDH* (c.1106G>A, p.Gly369Asp; c.553G>A, p.Glu185Lys). Labs and echocardiogram are normal, and the patient is being monitored as an affected case.

CPT II deficiency (4)	Four patients were diagnosed with CPT II deficiency (1 female, 3 males). Two of these patients died at 31 days and 14 years of age, respectively. The first patient's NBS result became available on day 10 of life; unfortunately, the patient passed away in the NICU at 31 days of age due to metabolic decompensation, compounded by mismanagement of the condition. The second patient died from complications of COVID-19. Prior to his death, he had been clinically stable with effective management of CPT II deficiency.
CACT deficiency (1)	One patient was diagnosed with CACT deficiency via NBS. She was started on a high-carbohydrate, low long-chain fat diet, supplemented with MCT oil and carnitine. Despite these interventions, she developed dilated cardiomyopathy with progressively worsening cardiac function. Unfortunately, she passed away at age 2 due to cardiac failure.
LCHAD (1)	One patient was diagnosed with LCHAD deficiency. The patient passed away from an unknown cause at 8 days of life, on the same day the NBS result became available.
Cases missed by NBS (5)	
CPT II deficiency (2)	Two siblings, currently aged 13 and 18 years, had normal results on NBS. One sibling was diagnosed with CPT II deficiency at approximately 3 years of age during an evaluation for autism. He also had a history of recurrent episodes of elevated creatine kinase (CK). At the time of diagnosis, a plasma acylcarnitine profile was suggestive of CPT II deficiency. Following this diagnosis, his older brother, who also had a history of recurrent episodes of rhabdomyolysis, was subsequently diagnosed with the same condition at age 9 yr.
Citrin deficiency (1)	One patient, currently 4 y11m old, had normal results on NBS. At 5 months of age, the patient presented with persistent elevation of AST and ALT. A cholestasis gene panel revealed compound heterozygous pathogenic variants in *SLC25A13*. The patient is currently in the adaptation stage and remains clinically stable with dietary modifications. Her mean ± SD citrulline level was 36.7 ± 13.7 µmol/L (reference range: 7–40 µmol/L).
ASA deficiency (1)	One patient, currently 8y9m old, was initially considered a false positive due to a normal plasma amino acid profile, and the case was closed. However, at 2 years and 3 months of age, the patient was re-evaluated for developmental delay and autism. Repeat testing revealed elevated argininosuccinic acid (10 nmol/mL; reference <2 nmol/mL), and genetic analysis confirmed a diagnosis of ASA with compound heterozygous pathogenic variants in the ASL gene. Treatment was initiated with dietary protein restriction and arginine supplementation. The patient has never experienced metabolic decompensation but presents with language delays and autism.
Cobalamin C deficiency (1)	The patient (10 years, 7 months) was missed by NBS and diagnosed at 1 month of age after presenting at 1 week with hypothermia and failure to thrive. The mean ± SD total homocysteine was 27 ± 8 µmol/L. Global developmental delay was noted.

Note: Ages reported in the table reflect patient age at the time of data analysis.
